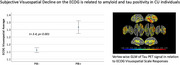# Subjective Visuospatial Decline in Cognitively Unimpaired Individuals

**DOI:** 10.1002/alz70857_106551

**Published:** 2025-12-26

**Authors:** Deepti Putcha, Michael J. Properzi, Kathryn V Papp, Keith A. Johnson, Reisa A. Sperling, Dorene M. Rentz, Rebecca E. Amariglio

**Affiliations:** ^1^ Frontotemporal Disorders Unit, Department of Neurology, Massachusetts General Hospital and Harvard Medical School, Boston, MA, USA; ^2^ Department of Neurology, Massachusetts General Hospital, Harvard Medical School, Boston, MA, USA; ^3^ Massachusetts General Hospital, Brigham and Women's Hospital, Harvard Medical School, Boston, MA, USA; ^4^ Departments of Neurology and Radiology, Massachusetts General Hospital, Harvard Medical School, Boston, MA, USA; ^5^ Massachusetts General Hospital, Brigham and Women's Hospital, Harvard Medical School, Boston, MA, USA; ^6^ Massachusetts General Hospital, Harvard Medical School, Department of Neurology, Boston, MA, USA; ^7^ Brigham and Women's Hospital, Harvard Medical School, Boston, MA, USA

## Abstract

**Background:**

The presence of Subjective Cognitive Decline (SCD) in cognitively unimpaired (CU) individuals represents a significant risk factor for progression from preclinical to the symptomatic stage of Alzheimer's disease (AD). Studies on SCD to date have focused on memory concerns as a risk factor for developing amnestic AD dementia. However, AD pathology underlies a heterogeneous phenotypic spectrum, including a visual variant of AD—Posterior Cortical Atrophy (PCA)—thought to comprise 5‐15% of AD dementia cases. We do not yet have a method for identifying individuals at the preclinical stage of AD who go on to develop PCA.

**Method:**

Self‐report responses on the Everyday Cognition Scale (ECOG) from 253 CU participants (mean age = 72.1 ± 8.9) in the Harvard Aging Brain Study were analyzed. We explored associations between total participant responses on the visuospatial subscale, objective cognitive tests, and amyloid PET positivity. An exploratory whole‐cortex tau PET general linear model was conducted to examine the association between subjective visuospatial decline and emerging tau burden in the neocortex.

**Result:**

Four percent of participants (*N* = 9) endorsed “at least occasional problems” or more averaged across the 7‐item ECOG visuospatial subscale. These individuals did not differ from the rest of the sample on age, sex, education, or MMSE. ECOG visuospatial scores across the whole CU group were unrelated to MMSE or objective visuospatial cognition. ECOG visuospatial scores were higher in PiB+ individuals compared to PiB‐ individuals (*t* = 3.4, *p* = 0.001). Subjective visuospatial concerns were associated with right‐hemisphere predominant tau in temporoparietal and prefrontal cortices, largely overlapping with regions associated with subjective memory concerns and with tau epicenters reported in preclinical AD.

**Conclusion:**

A subset of CU individuals endorsed subjective visuospatial decline, a cognitive domain that does not typically decline in healthy aging. The positive relationships observed between subjective visuospatial decline and biomarkers of amyloid and tau suggest that the ECOG can be a useful tool in capturing subtle visuospatial decline in addition to early memory concerns in preclinical AD. Developing methodology to predict the development of atypical AD variants has significant implications for optimizing early diagnosis and treatment of this disease.